# Targeted Demethylation of the PLOD2 mRNA Inhibits the Proliferation and Migration of Renal Cell Carcinoma

**DOI:** 10.3389/fmolb.2021.675683

**Published:** 2021-06-09

**Authors:** Congcong Cao, Qian Ma, Xinbo Huang, Aolin Li, Jun Liu, Jing Ye, Yaoting Gui

**Affiliations:** ^1^ Guangdong and Shenzhen Key Laboratory of Male Reproductive Medicine and Genetics, Institute of Urology, Peking University Shenzhen Hospital, Shenzhen-Peking University-The Hong Kong University of Science and Technology Medical Center, Shenzhen, China; ^2^ Department of Urology, Peking University First Hospital, Beijing, China

**Keywords:** CRISPR, dCas13b, (N6-methyladenosine), PLOD2, renal cell cancer

## Abstract

N6-methyladenosine (m^6^A) RNA modification is the most common internal mRNA modification in mammals and has been reported to play a key role in gene expression regulation. In this study, we detected a high level of m^6^A methylation of the PLOD2 3′-untranslated regions (3′UTR) in renal cell carcinoma (RCC). Furthermore, we found that the high expression level of PLOD2 was a prognostic indicator for patients with RCC. A dm^6^ACRISPR demethylation system was performed to accurately and specifically demethylate 3′UTR of PLOD2 and caused an inactivation of PLOD2 expression. Furthermore, we also performed many *in vitro* experiments to confirm that PLOD2 exerted tumor promoter effects by promoting tumor proliferation and migration. In conclusion, PLOD2 mRNA demethylated by dCas13b-ALKBH5 might provide a new light on the treatment for RCC.

## Introduction

Renal cell carcinoma (RCC) is one of the most common adult genitourinary cancers. According to the latest cancer statistics report, there are more than 65,000 newly diagnosed cases in the United States each year, and nearly 15,000 of them have renal cancer-related deaths, making it the sixth most common tumor site (Siegel, Miller, and Jemal 2019). Renal clear cell carcinoma (ccRCC) is the most common histologic subtype of renal cell carcinoma, accounting for 70% (Ronald and Bukowski. 1997). Although diagnostic techniques for renal cell carcinoma have improved in recent decades, ∼30% of patients with RCC have metastatic or local progression at the time of diagnosis. Since ccRCC is resistant to chemoradiotherapy, surgery is still an effective method for the treatment of ccRCC. However, about one-third of patients with localized renal cancer develop local recurrence and metastasis after surgical treatment. Therefore, there is an urgent need to explore the molecular mechanism of renal cell carcinoma and find new effective targets for treatment and intervention of patients with renal cell carcinoma.

N6-methyladenosine (m^6^A) RNA modification is an epigenetic modification that occurs at the transcriptome level, and is the most common post-transcriptional modification of mRNA in eukaryotes. It is widely involved in a series of RNA-related metabolic activities such as RNA stability regulation, splicing processing, transport and translation (Dominissini et al., 2012; Meyer et al., 2012; Bodi et al., 2015). About 1/3 of human gene transcripts are m^6^A modified, with an average of 3–5 m^6^A modifications per mRNA. M^6^A modification is a dynamic and reversible process that is mainly regulated by three types of proteins: RNA methyltransferases (Writers), which promote the methylation of N6-adenylate in RNA, including Mettl3, Mettl14, and WATP; RNA demethylases (erasers) were used to remove N6-adenosine methylation, and FTO and ALKBH5 were common (Jia et al., 2011; Liu et al., 2014; Ping et al., 2014; Schwartz et al., 2014). M^6^A recognition proteins (readers) are responsible for binding to m^6^A sites on RNA to affect splicing (Dominissini et al., 2012), processing (Wang et al., 2014), degradation and other processes of RNA, mainly including YTHDFs and YTHDCs subtypes (Deng et al., 2018; Huang, Weng, and Chen 2020).

PLOD2 is a member of the PLOD (Procollagen-Lysine, 2-oxoglutarate 5-dioxygenase) family, which selectively hydroxylates the lysine residues of collagen-terminal peptides to promote the picoline-hydroxyl cross-linking reaction of collagen, thereby enhancing the stability of collagen (Gilkes et al., 2013). Collagen is the most abundant protein in the extracellular matrix, which is known as the “highway” for tumor cell migration and invasion. Semenza et al.’s study showed that hypoxic inducible factor 1 (HIF-1) can induce the expression of collagen hydroxylase P4HA1, P4HA2, and PLOD2 under hypoxic conditions, change extracellular matrix fiber arrangement, and then affect cell morphology, adherent ability and directional migration ability (Eisinger-Mathason et al., 2013). PLOD2 expression was up-regulated in various malignant tumors and negatively correlated with prognosis. It has been reported that in sarcoma (Miyamoto et al., 2016), pancreatic cancer (Kurozumi et al., 2016) and breast cancer (Dong et al., 2005), HIF-1α can regulate the expression of PLOD2 and thus affect tumor migration and invasion ability (Rajkumar et al., 2011). PLOD2 up-regulates the expression of HK2 through the STAT3 signaling pathway, which is a key enzyme in glycolysis, thereby affecting the proliferation, migration and invasion of colorectal cancer cells (Reis et al., 2011). PLOD2 knockdown in glioma can inhibit the PI3K/Akt signaling pathway, and then inhibit the expression of downstream EMT-related molecules such as E-cadherin and Snailin (Blanco et al., 2012). Recent studies have shown that in head and neck squamous cell carcinoma (HNSCC), integrin β1 is more stable after Plod2-hydroxylated modification, and is subsequently recruited to the cell membrane to regulate tumor cell movement, thereby promoting tumor invasion and metastasis (Dong et al., 2005).

In this research, we found that the PLOD2 m^6^A modification of mRNA and expression levels were significantly elevated in RCC. Then we wanted to clarify the effect of PLOD2 in the development of renal cancer and its molecular mechanism of m^6^A methylation modification. Based on previous literature investigation and preliminary experimental results, we speculated that m^6^A modification of PLOD2 could promote the translation of PLOD2 protein after YTHDF1 recognition, and then activate key molecules in the signaling pathway related to migration and invasion, thus promoting the occurrence and development of renal cancer.

In this study, we can achieve site-specific regulation of m^6^A modification on PLOD2 mRNA by transfected with CRISPR/dCas13b-ALKBH5 and targeted PLOD2 sgRNAs (Li et al., 2020). At the cell level, the effects of PLOD2 m^6^A modification on the proliferation, migration, invasion and apoptosis of renal carcinoma cells were analyzed to explore its molecular mechanism in the occurrence and development of renal carcinoma.

## Results

### The Expression Level and N6-Methyladenosine Methylation Level of PLOD2 Were Significantly Increased in Renal Cell Carcinoma Tissues

**FIGURE 1:**
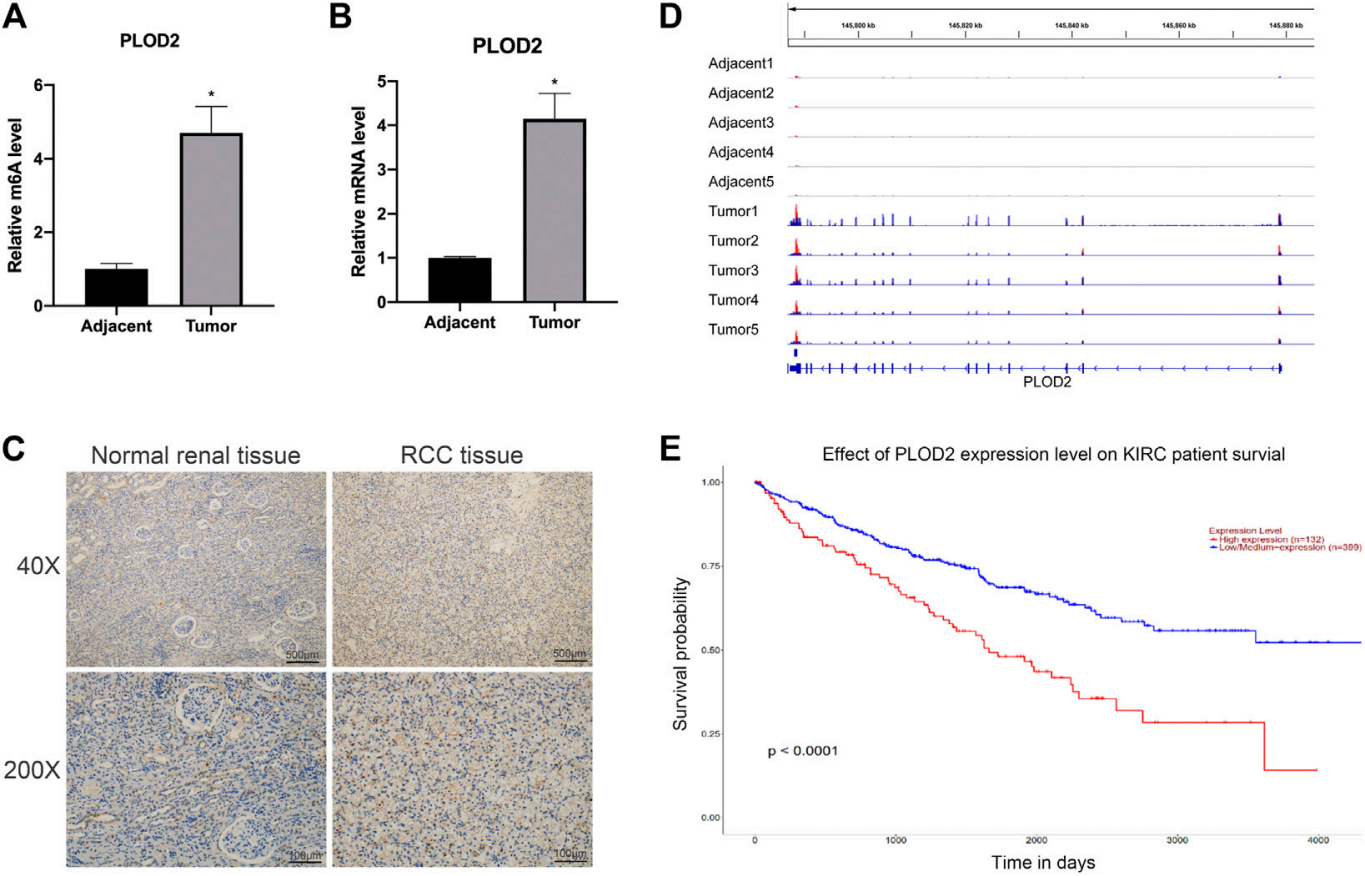
The mRNA and m6A methylation levels of PLOD2 were significantly increased in RCC tissues compared to adjacent tissues. **(A)** M^6^A enrichment of PLOD2 mRNA in five pairs of RCC tissues and matched adjacent tissues was measured by MeRIP-qPCR analysis. **(B)** PLOD2 mRNA expression levels in tumor and adjacent tissues were detected by RT-qPCR. **(C)** Representative IHC stains of PLOD2 in RCC tumor and adjacent tissues. **(D)** The m^6^A abundances on PLOD2 mRNA transcripts in five pairs of tumor and adjacent tissues by m^6^A-RIP-seq. **(E)** Survival analysis of PLOD2 expression in RCC patients. *p < 0.05.

To study the functional role of PLOD2 m^6^A methylation in renal cell carcinoma (RCC), the PLOD2 m^6^A methylation levels were measured in five tumor tissues relative to their normal control samples. A significant increase in m^6^A modification in PLOD2 mRNA was observed in tumor samples (Figure 1A). Moreover, our previous results exhibited that m^6^A methylation of PLOD2 in 3′’-untranslated regions increased remarkably in five tumor tissues compared to their paired adjacent normal tissues (Figure 1D). Then we checked the expression levels of PLOD2 in RCC tumor and adjacent tissues by RT-qPCR and IHC. We found the mRNA and protein levels of PLOD2 were significantly increased in RCC tumor tissues compared to adjacent tissues (Figures 1B,C). Besides, RCC patients with higher PLOD2 expression obtained shorter overall survival (OS) from TCGA database (Figure 1E). These results implied that dysregulation of PLOD2 mRNA and m6A methylation level might be involved in the progression of RCC.

### The mRNA and N6-Methyladenosine Methylation Level of PLOD2 Was Significantly Upregulated in Renal Cell Carcinoma Cell Lines

**FIGURE 2:**
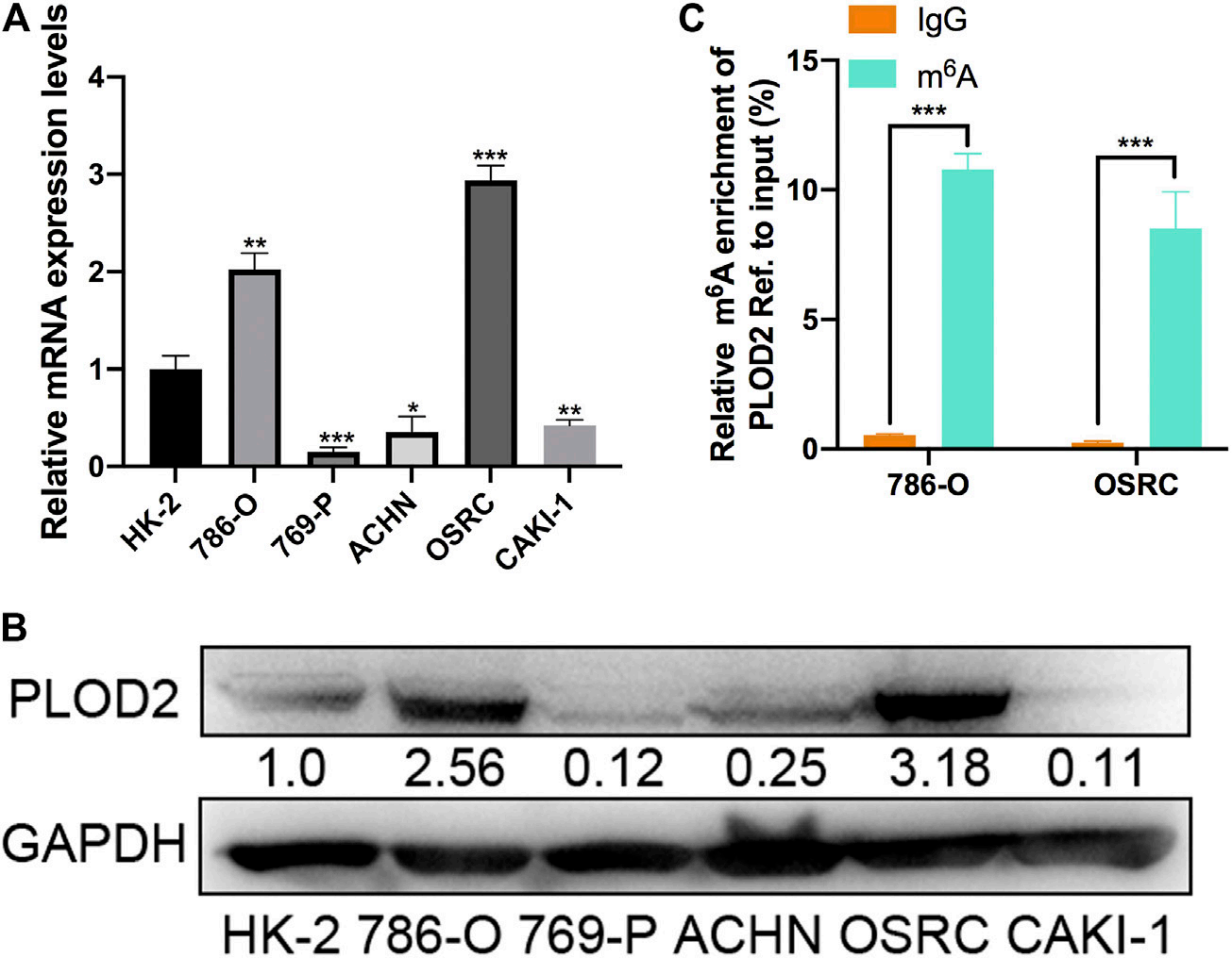
The mRNA and m^6^A methylation levels of PLOD2 were obviously increased in RCC cell lines. **(A,B)** PLOD2 mRNA and protein expressions in RCC cell lines (786-O, 769-P, ACHN, OSRC, and CAKI-1) and normal epithelium cells of renal tubule HK2 by RT-qPCR and western blot. **(C)** m^6^A enrichment of PLOD2 in 786-O and OSRC RCC cells detected by MeRIP-qPCR. *p < 0.05, **p < 0.01, ***p < 0.005.

To validate the expression pattern of PLOD2 *in vitro*, we firstly measured the mRNA and protein level of PLOD2 in RCC cell lines (786-O, 769-P, ACHN, OSRC, and CAKI-1) by RT-qPCR and western blotting. We found PLOD2 was highly expressed in 786-O and OSRC RCC cell lines compared to HK-2, a normal renal tubule epithelium cell (Figures 2A,B). Therefore, we then examined the m^6^A methylation levels of PLOD2 in 786-O and OSRC cells which PLOD2 was highly expressed. The results showed that PLOD2 mRNA was highly methylated measured by MeRIP-qPCR in 786-O and OSRC cells (Figure 2C).

### dm^6^ACRISPR Induced Demethylation of PLOD2 mRNA

**FIGURE 3:**
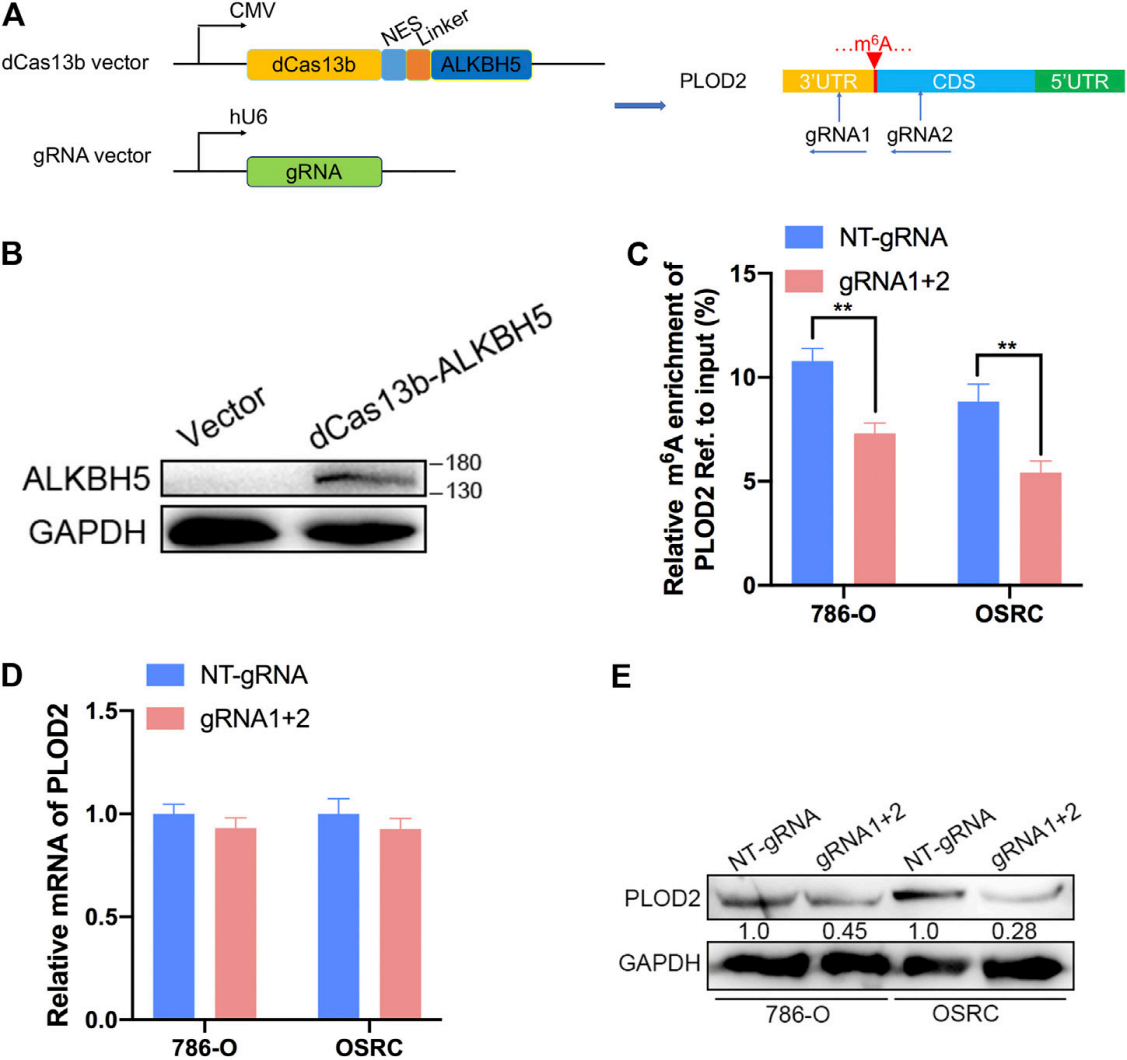
dm^6^ACRISPR induced demethylation of PLOD2 mRNA. **(A)** Construction of dCas13b-ALKBH5 with two gRNAs targeting methylation region of PLOD2 3’UTR. **(B)** dCas13b-ALKBH5 fusion protein was examined in cell by western blot. **(C)** MeRIP-qPCR analysis of PLOD2 mRNA in 786-O and OSRC RCC cells after transfected with dCas13b-ALKBH5 and sgRNAs for 24 h. **(D)** mRNA expression levels of PLOD2 in 786-O and OSRC RCC cells after transfected with dCas13b-ALKBH5 and sgRNAs for 24 h by RT-qPCR. **(E)** Protein levels of PLOD2 in 786-O and OSRC RCC cells after transfected with dCas13b-ALKBH5 and sgRNAs for 24 h by western blot. **p < 0.01.

Then, PLOD2 mRNA was specifically demethylated by transfected with CRIPSR/dCas13b-ALKBH5 and PLOD2 sgRNAs in RCC cells lines. We designed two gRNAs targeted PLOD2 mRNA methylation region (Figure 3A). Firstly, dCas13b-ALKBH5 fusion protein was examined in cell by western blot (Figure 3B). Next, the demethylation effect of dm^6^ACRISPR on PLOD2 mRNA was validated by MeRIP-qPCR. We found m^6^A levels of PLOD2 mRNA were significantly inhibited after transfected with dCas13b-ALKBH5 and PLOD2 sgRNAs in 786-O and OSRC cells (Figure 3C). However, there were no significant change of PLOD2 mRNA levels after transfected with dCas13b-ALKBH5 and PLOD2 sgRNAs (Figure 3D). But the protein levels of PLOD2 were decreased after transfected with dCas13b-ALKBH5 and PLOD2 sgRNAs in 786-O and OSRC cells (Figure 3E). In conclusion, our results exhibited that dCas13b-ALKBH5 could decrease protein level not mRNA level of PLOD2 after demethylating its mRNA.

### Targeting Demethylation of PLOD2 Transcripts by dCas13b-ALKBH5 Inhibited Renal Cell Carcinoma Cells Proliferation

**FIGURE 4:**
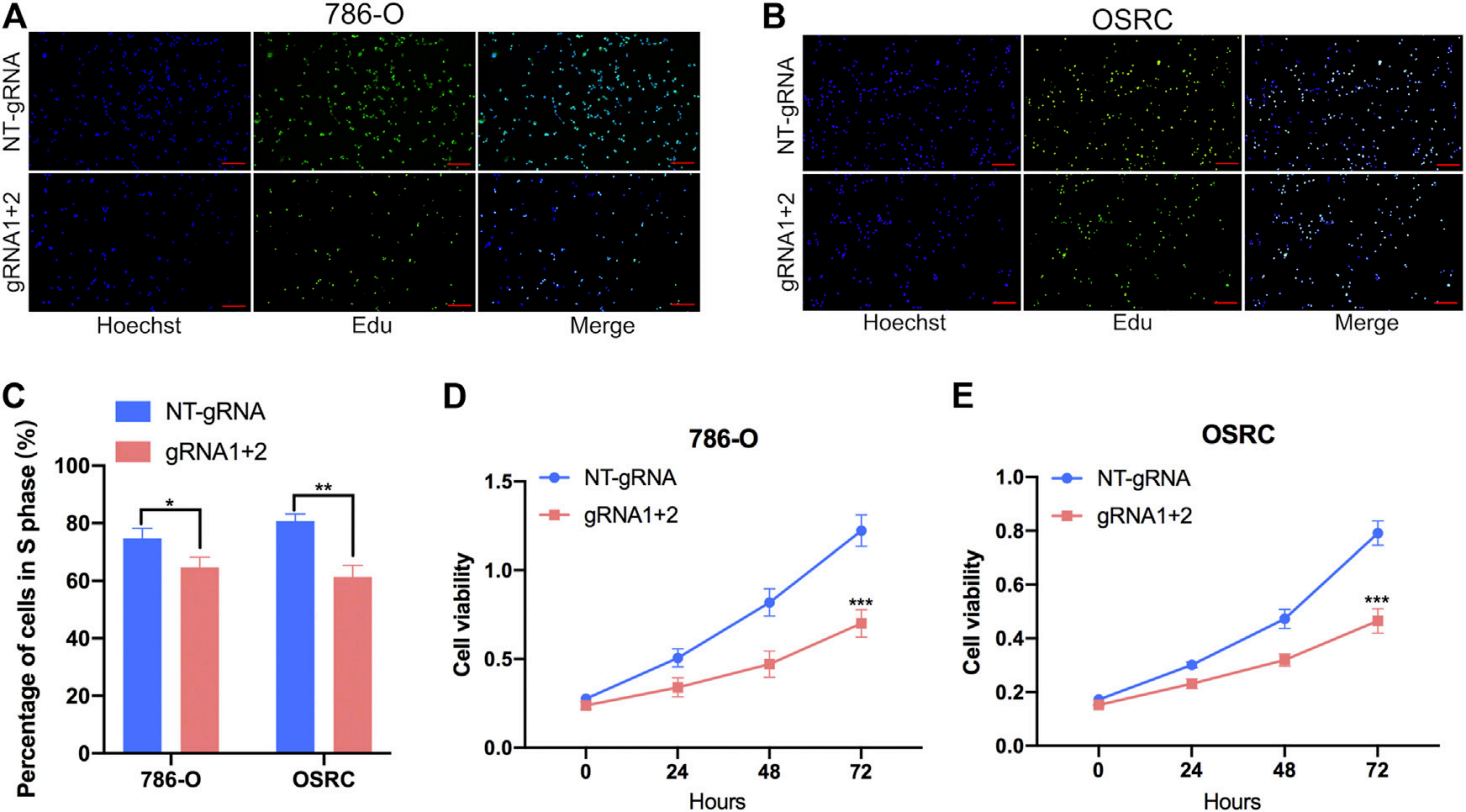
Targeting demethylation of PLOD2 transcripts by dCas13b-ALKBH5 inhibited RCC cells proliferation. **(A–C)** EdU assays of 786-O and OSRC cells after transfected with dCas13b-ALKBH5 and sgRNAs. **(D,E)** CCK-8 assays of 786-O and OSRC cells after transfected with dCas13b-ALKBH5 and sgRNAs. *p < 0.05, **p < 0.01, ***p < 0.005.

To further investigate that dm^6^ACRISPR targeting PLOD2 whether can modulate RCC cells proliferation, we performed 5-ethynyl-20-deoxyuridine (EdU) and cell counting kit-8 (CCK-8) assays to evaluate the proliferative activity of RCC cells after dm^6^ACRISPR targeting PLOD2 in 786-O and OSRC RCC cells. And the results showed that demethylation of PLOD2 mRNA induced by dm^6^ACRISPR led to a decreased cell proliferation rate in the 786-O and OSRC RCC cells as compared with that of non-targeted gRNA combined with dCas13b-ALKBH5 (Figures 4A–E). These results confirmed that targeted demethylation of PLOD2 transcripts could inhibit RCC cells proliferation.

### Targeting Demethylation of PLOD2 Transcripts by dCas13b-ALKBH5 Inhibited Renal Cell Carcinoma Cells Migration

**FIGURE 5:**
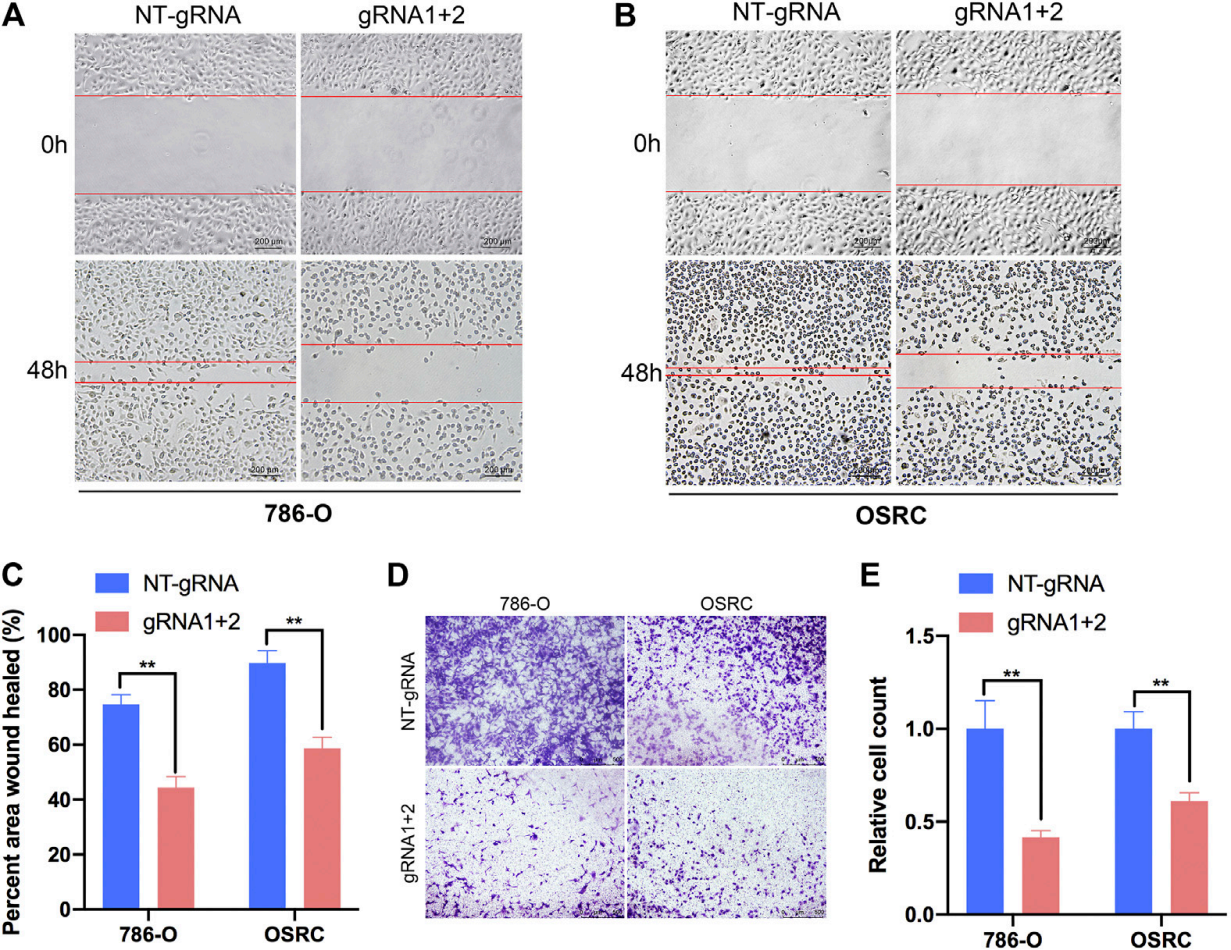
Targeting m^6^A of PLOD2 transcripts by dm^6^ACRISPR inhibited RCC cells migration. **(A–C)** Wound healing assays of 786-O and OSRC cells after transfected with dCas13b-ALKBH5 and sgRNAs. **(D,E)** Transwell migration assays of 786-O and OSRC cells after transfected with dCas13b-ALKBH5 and sgRNAs. **p < 0.01.

To further explore the role of m^6^A methylation of PLOD2 in RCC migration, we demethylated PLOD2 mRNA by dm^6^ACRISPR in 786-O and OSRC RCC cells. Notably, an obvious delay in migration was observed in PLOD2-demethylating RCC cells in a scratch wound healing assay (Figures 5A–C). Furthermore, PLOD2 demethylation significantly suppressed the migration of RCC cells in transwell assay (Figures 5D,E). These results confirmed that targeted demethylation of PLOD2 transcripts in RCC cells inhibited wound closure and migration activity.

### YTHDF1 Involved in N6-Methyladenosine Regulated Expression of PLOD2

**FIGURE 6:**
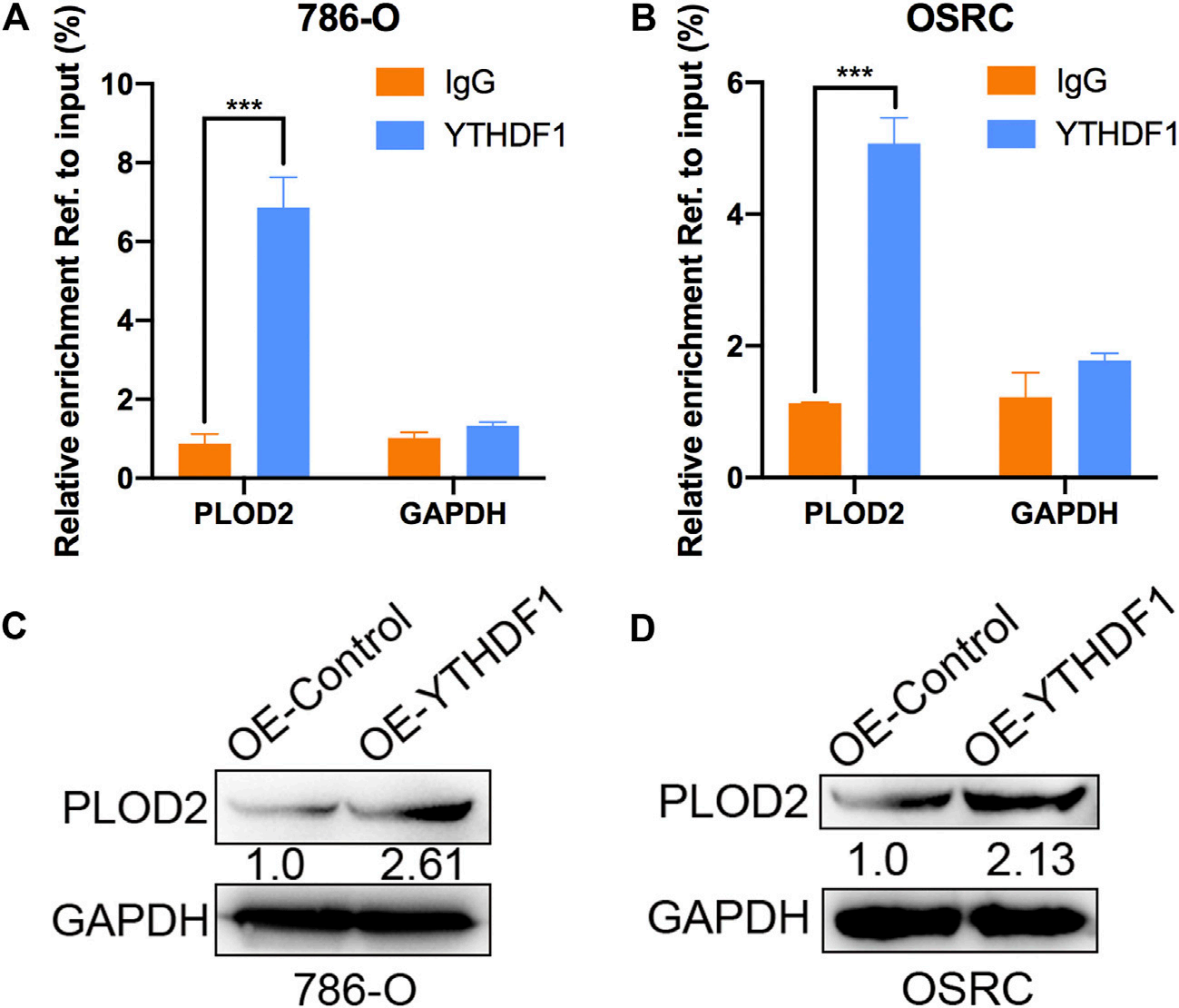
YTHDF1 involved in m^6^A regulated protein level of PLOD2. **(A,B)** YTHDF1 RIP-qPCR analysis of PLOD2 mRNA in 786-O and OSRC cells. GAPDH as a negative control. **(C,D)** Western blot analysis of PLOD2 protein levels in 786-O and OSRC cells transfected with control or YTHDF1 construct for 24 h. ***p < 0.005.

Mechanism of targeting demethylation of PLOD2 transcripts by dCas13b-ALKBH5 inhibited its protein level need to be further investigated. YTHDF1 acted as “reader” can recognize m^6^A methylated mRNA and regulate the methylated mRNA translation (Xiao et al., 2015). We then examined if there was relationship between YTHDF1 and methylated PLOD2 mRNA by RIP-qPCR. The results showed that PLOD2 mRNA could be interacted by YTHDF1 protein in 786-O and OSRC RCC cells (Figures 6A,B). In addition, after overexpressed YTHDF1 in 786-O and OSRC RCC cells, we found the protein levels of PLOD2 were increased which confirmed previous data (Figures 6C,D). These results validated that YTHDF1 could interact with methylated PLOD2 mRNA and regulate its protein level by m^6^A modification.

### Targeted Demethylation of PLOD2 mRNA Regulated Downstream Target Genes Expression

**FIGURE 7:**
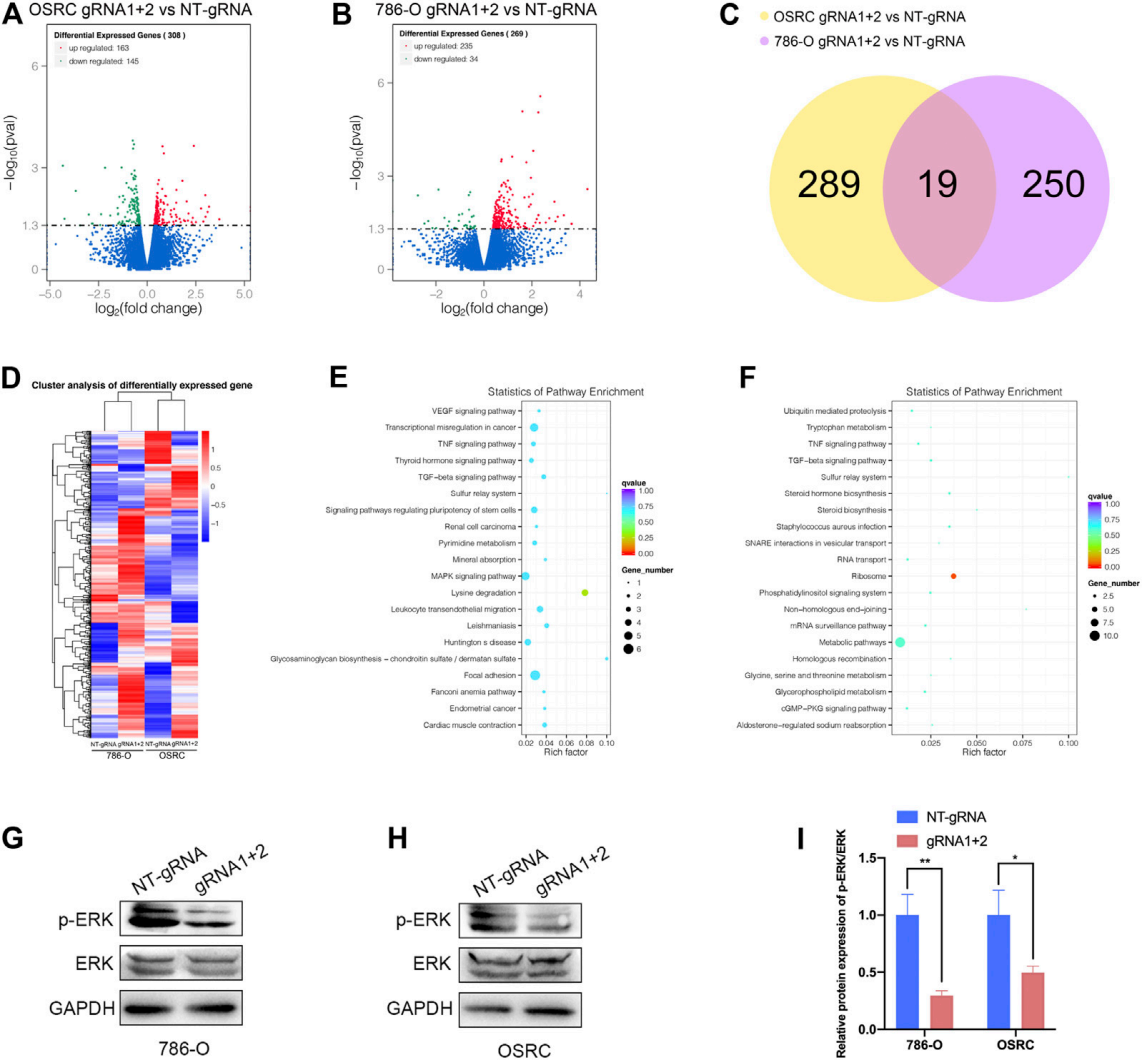
Targeted demethylation of PLOD2 mRNA regulates downstream target genes expression. **(A,B)** The scatter diagram showed the differentially expressed genes (DEGs) in PLOD2 demethylated 786-O and OSRC cells. **(C)** The venn diagram summarized the overlap of PLOD2 regulated genes in both cells. **(D)** The heatmap showed the differentially expressed genes (DEGs) in PLOD2 demethylated 786-O and OSRC cells. **(E,F)** KEGG pathway analysis of DEGs in 786-O and OSRC cells. **(G–I)** The protein levels of MAPK pathway related proteins were measured in PLOD2 demethylated 786-O and OSRC cells by western blot.

Finally, we examined the impact of PLOD2 mRNA demethylation on the transcriptome of 786-O and OSRC RCC cells by RNA-seq. We identified 308 and 269 differentially expressed genes in PLOD2 demethylated OSRC and 786-O cells compared to control cells (Figures 7A,B,D). By comparing differentially expressed genes in two cell lines, we identified 19 genes that might be regulated by PLOD2 (Figure 7C). These differentially expressed genes were further categorized based on the KEGG pathway, and we found they were associated with “Lysine degradation,” “VEGF signaling pathway,” “Transcriptional misregulation in cancer,” “Renal cell carcinoma,” “MAPK signaling pathway,” “TNF signaling pathway,” and “Metabolic pathways” in two RCC cell lines related with PLOD2 demethylation (Figures 7E,F). Finally, we demonstrated that demethylation of PLOD2 inhibited the activation of the MAPK signaling pathway in RCC cells (Figures 7G–I).

## Discussion

Renal cell carcinoma (RCC) is one of the most common malignancies of urinary system. In 2015, it was reported that there were 66,800 new cases and 23,400 deaths from renal cell carcinoma in China (Siegel, Miller, and Jemal 2019). Clear cell renal cell carcinoma (CCRCC) is the main pathological type of renal cell carcinoma, accounting for 70–80% of renal cell carcinoma (Ronald and Bukowski 1997). RCC has no specific clinical manifestations and features in its early stage, and 20–30% have metastasized at the time of initial diagnosis. Nephrectomy is the main treatment for renal cell carcinoma (RCC). The 5 years survival rate for patients with early stage surgery is more than 80%, while the prognosis for patients with advanced metastasis is very poor. Renal cell carcinoma is not sensitive to radiotherapy and chemotherapy. Existing targeted drugs have uncertain efficacy, and most of them are still in clinical trials. Therefore, further study of the molecular mechanism of the occurrence and development of renal cell carcinoma and search for specific diagnostic markers and therapeutic targets are of great academic significance and application value for the early diagnosis and precise treatment of renal cell carcinoma.

N6-methyladenosine RNA modification on the sixth nitrogen atom of RNA molecule adenine has become one of the hot topics in the field of biomedical research in recent years (Dominissini et al., 2012; Meyer et al., 2012; Bodi et al., 2015). More and more studies have shown that abnormal m^6^A modification is closely related to the occurrence and development of tumors (Jia et al., 2011; Liu et al., 2014; Ping et al., 2014; Schwartz et al., 2014). This project used MeRIP-seq to analyze the differences in m^6^A methylation modification profiles of five pairs of RCC tumor tissues and adjacent tissues, and combined with RNA-seq to compare the differences in gene expression profiles of tumor tissues and adjacent tissues. Through joint analysis, a number of genes that might be caused by m^6^A modification changes were found to change the expression levels. Among them, the modification and expression level of m^6^A on PLOD2 mRNA were significantly increased in renal clear cell carcinoma tissues, which aroused our research interest.

Our preliminary experimental results showed that the incidence of tumor metastasis was significantly increased in RCC patients with elevated PLOD2 expression in cancer tissues, and overall survival rate was significantly reduced. And demethylation of PLOD2 significantly inhibited the proliferation and migration of RCC cells, suggesting that PLOD2 played an important role in the development and progression of RCC. Overexpression of YTHDF1 in RCC cells significantly up-regulated the protein levels of PLOD2, suggesting that modification of m^6^A could regulate the protein level of PLOD2. Finally, the RNA-seq data showed that typical pathways involved in RCC development and progression such as “Lysine degradation,” “VEGF signaling pathway,” “Transcriptional misregulation in cancer,” “Renal cell carcinoma,” “MAPK signaling pathway,” “TNF signaling pathway,” and “Metabolic pathways” were enriched by KEGG pathway analysis. These results demonstrate that PLOD2 is a transcriptional regulator associated with cancer development and progression.

This study explores the role and molecular mechanism of PLOD2 m^6^A modification in the occurrence and development of RCC through targeted regulation of PLOD2 mRNA methylation by dm^6^ACRISPR. The implementation of this study will have important academic value in further elucidating the molecular mechanism of the occurrence and development of renal cell carcinoma, and will also provide a new way for the early diagnosis and precise treatment of renal cell carcinoma.

## Methods

### Patients and Tissue Specimen Collection

Five primary clear cell renal cell carcinoma (ccRCC) samples and five adjacent non-malignant renal tissues with patients’ informed consent were obtained from the Urology Department of Peking University First Hospital (PKUFH), Beijing, China. This study followed the Helsinki declaration and was approved by the Institutional Ethical Review Board of PKUFH. The pathological diagnosis was made by professional urological pathologists. Samples were collected immediately in the operating room after surgical removal and were stored in liquid nitrogen after rapid freezing in liquid nitrogen for the following RNA isolation. We used these samples for the later mRNA and protein analysis.

### RT-qPCR and MeRIP-qPCR

Total RNA of primary RCC tissues and cell lines were extracted using an RNA-easy Isolation Reagent (Vazyme Biotech, Nanjing, China) according to their instructions, respectively, as previously described. The fragmented RNA was incubated with anti-m^6^A antibody-coupled beads. The m^6^A-containing RNAs were then immunoprecipitated and eluted from the beads. Both input control and m^6^A-IP samples were subjected to RT-qPCR with gene-specific primers. cDNA was synthesized using HiScript III RT SuperMix for qPCR (Vazyme Biotech, Nanjing, China). qRT-PCR was performed using spectrophotometry (ABI Prism 7500TM instrument, Applied Biosystems) with universal SYBR Green qPCR Master Mix (Vazyme Biotech, Nanjing, China). Glyceraldehyde 3-phosphate dehydrogenase (GAPDH) was used as reference gene. Primers were listed in Supplementary Table S1.

### Vector Construction and Design of gRNAs

The CRISPR dCas13b plasmids and Cas13b-gRNA plasmids were purchased from Addgene. And All designed gRNAs and dCas13b-ALKBH5 vector were constructed by Synbio Technologies Company (Suzhou, China). The sequences of related plasmids were listed in Supplementary Table S1.

### Cell Culture and Plasmid Transfection

RCC cell lines (786-O, 769-P, ACHN and OSRC) were used in this study. HK-2 human kidney proximal tubular epithelial cells were used as normal controls. These cell lines were purchased from the American Type Culture Collection (ATCC, Manassas, VA, United States) and National Infrastructure of Cell Line Resource, China. Cell lines were routinely cultured in RPMI 1640 or DMEM, which was supplemented with 10% fetal bovine serum (Invitrogen, Carlsbad, CA, United States) and incubated in a 5% CO_2_ environment at 37°C. All plasmids were transfected with lipo3000 (Invitrogen) following manufacture’s protocol and the number of plasmids used in the experiments were 1 μg.

### Protein Isolation and Western Blot

Total protein of cells was extracted by KeyGEN Bio TECH protein extraction kit (KGP1100) and separated on 10% SDS-PAGE and transferred onto nitrocellulose membrane. After blocking, blots were immunostained with primary antibodies and secondary antibodies respectively as previously described. The antibodies were as follows: PLOD2 (1:1,000; Preteintech, United States); ALKBH5 (1:1,000; Sigma, United States); YTHDF1 (1:1,000; Preteintech, United States); ERK (1:1,000; Cell Signaling Technology, United States); *p*-ERK (1:1,000; Cell Signaling Technology, United States) and GAPDH (1:10,000; Preteintech, United States). Immunohistochemistry staining was performed using a primary antibody of PLOD2 at a 1:300 dilution following a protocol described previously. All photographs were taken randomly and measured using Image Pro Plus (Media Cybernetics, Rockville, MD, United States).

### Wound-Healing Assay

The cell motility was assessed by scratch wound healing assay. 786-O and OSRC cells (2–3 × 106 per well) were plated in a six- well plate for 1 day and then transfected with vectors for 24 h. The cell layers were washed with PBS after carefully scratching by sterile tips. After incubation for 0 and 24 h, photos were taken. The assays were performed in triplicate.

### Transwell Migration Assay

The 786-O and OSRC cells suspended in 150 ul serum-free medium (2 × 105 cells/ml) were placed on the upper layer of a cell permeable membrane. Following another 24–48 h incubation, the cells migrated through the membrane were stained with 1% Crystal Violet and counted.

### RNA-Sequencing

Total RNA was prepared from RCC cells after transfected with the dCas13b-ALKBH5 and gRNAs using Rneasy kit (Qiagen) and was subjected to RNA-seq according the mRNA sequencing protocol provided by Illumina (TruSeq RNA Sample Preparation Kit). In brief, poly(A)-containing mRNA molecules were purified using poly(T)-oligo-attached magnetic beads, fragmented and applied to first-strand complementary DNA (cDNA) synthesis using reverse transcriptase and random primers. Second-strand cDNA synthesis was performed using DNA polymerase I and RNase H. cDNAs were then end-repaired, A-tailed, ligated to adaptors and amplified to create the final cDNA library. Afterwards, adaptor-ligated cDNA was sequenced on a HiSeq2000 sequencer according to the manufacturer’s instructions.

### Statistical Analysis

When comparing two groups of measurement data, t test was used for data conforming to normal distribution, whereas a Wilcoxon test was used for data not conforming to normal distribution, and the measurement data were expressed as the mean ± standard deviation (SD). A Chi-square test was used to analyze comparisons between groups for enumeration data. The Kaplan-Meier method was used for survival analysis, and a log-rank test was applied for comparations between groups. R packages used in this study included “GDCRNATools,” “clusterProfiler,” “org.Hs.eg.db,” “tidyr,” “dplyr,” “ggplot2,” “ggsignif,” “survival,” and “survimier”. Annotation gene sets used in GSEA were hallmark gene sets from the Molecular Signatures Database (MSigDB). All statistical analyses were performed and visualized using RStudio (Version1.2.1335, Boston, MA, United States), GSEA (Version4.0, UC San Diego and Broad Institute, United States) 23, Medcalc (Version16.8, Ostend, Belgium), and GraphPad Prism (Version 8.0, GraphPad, Inc., La Jolla, CA, United States). A two-tailed *p* < 0.05 was considered statistically significant.

## Data Availability

The data presented in the study are deposited in the (NCBI SRA) repository, accession number (PRJNA718295).
